# Clinical Significance of Early Carcinoembryonic Antigen Change in Patients With Nonmetastatic Colorectal Cancer

**DOI:** 10.3389/fonc.2022.739614

**Published:** 2022-05-09

**Authors:** Younghoo Jo, Jae-Hoon Lee, Eun-Suk Cho, Hye Sun Lee, Su-Jin Shin, Eun Jung Park, Seung Hyuk Baik, Kang Young Lee, Jeonghyun Kang

**Affiliations:** ^1^ Yonsei University College of Medicine, Seoul, South Korea; ^2^ Department of Nuclear Medicine, Gangnam Severance Hospital, Yonsei University College of Medicine, Seoul, South Korea; ^3^ Department of Radiology, Gangnam Severance Hospital, Yonsei University College of Medicine, Seoul, South Korea; ^4^ Biostatistics Collaboration Unit, Yonsei University College of Medicine, Seoul, South Korea; ^5^ Department of Pathology, Gangnam Severance Hospital, Yonsei University College of Medicine, Seoul, South Korea; ^6^ Department of Surgery, Gangnam Severance Hospital, Yonsei University College of Medicine, Seoul, South Korea; ^7^ Department of Surgery, Severance Hospital, Yonsei University College of Medicine, Seoul, South Korea

**Keywords:** CEA, colorectal neoplasm, survival, iAUC, prognosis

## Abstract

**Background:**

This study aimed to evaluate the prognostic significance of preoperative, postoperative, and trajectory changes in carcinoembryonic antigen (CEA) levels in patients with colorectal cancer (CRC).

**Methods:**

This retrospective study included patients who underwent surgical resection for nonmetastatic CRC. The optimal cutoff values of preoperative CEA (CEA-pre), early postoperative CEA (CEA-post), and CEA level change (CEA-delta) were determined to maximize the differences in overall survival (OS) among groups. The patients were divided into three groups according to CEA-trend: normal, low CEA-pre; normalized, high CEA-pre/low CEA-post; elevated, high CEA-pre/high CEA-post. The integrated area under the curve (iAUC) was used to compare the discriminatory power of all variables.

**Results:**

A total of 1019 patients diagnosed with stage I–III CRC were enrolled. The optimal cutoff values of CEA level were determined as 2.3 ng/mL for CEA-pre, 2.3 ng/mL for CEA-post, and -0.93 ng/mL for CEA-delta. Although subgroup dichotomization showed that CEA-pre, CEA-post, CEA-delta, and CEA-trend were all associated with OS in univariate analysis, CEA-trend was the only independent prognostic factor in multivariate analysis. The iAUC of CEA-trend was superior to that of CEA-pre, CEA-post, and CEA-delta. Compared with the normal group, the normalized group showed worse OS (*p*=.0007) in stage II patients but similar OS (*p*=.067) in stage III patients.

**Conclusion:**

The optimal cutoff value of CEA level in the preoperative and postoperative periods was determined to be 2.3 ng/mL, and the combination of CEA-pre and CEA-post showed better prognostic stratification. However, its prognostic significance may differ depending on the CRC stage.

## Introduction

Carcinoembryonic antigen (CEA) has been shown to be associated with long-term survival outcomes in patients with various types of cancer, including colorectal cancer (CRC) (1–12). The normal range of CEA level has been considered to be 0–5 ng/mL, and many studies have used this criterion to predict the prognosis of patients. Although most studies assessed preoperative CEA levels (CEA-pre) or postoperative CEA levels (CEA-post) individually, (5–12) several systematic reviews on CEA levels’ ability to detect CRC recurrence argues that single CEA measurement is not a sufficient index, and the slope of the linear regression line of post-operational CEA showed better diagnostic performance (13, 14).

However, studies investigating the clinical significance of combination of preoperative and postoperative CEA (CEA-trend) in patients with CRC have shown conflicting results (15, 16). Konishi et al. reported that the persistently elevated group (high CEA-pre and high CEA-post) had significantly lower recurrence-free survival (RFS) than the normal (normal CEA-pre; hazard ratio [HR], 2.56; 95% confidence interval [CI], 1.44–4.52; *p*=.001) and normalized (high CEA-pre and low CEA-pre; HR, 2.43; 95% CI, 1.21–4.89; *p*=.02) groups. In addition, the 3-year RFS of the normalized group was similar to that of the normal group (HR, 1.05; 95% CI, 0.63–1.76; *p*=.85) in 1027 patients with colon cancer (15). Meanwhile, Nakamura et al. reported that the normalized group had worse overall survival (OS) than the normal group (*p*=.0001) in patients with rectal cancer. (16) The reason for these different observations is unclear. The significance of CEA-trend has been mainly evaluated using 5 ng/mL as the cutoff value of CEA level. Several studies have investigated the clinical significance of newly defined cutoff values of CEA level and suggested optimal cutoff values of 2.1 ng/mL, (17) 2.5 ng/mL, (18) and 3.5 ng/mL (19). In this regard, it remains unclear whether there is a difference in the strength of the association of CEA-trend with survival when using the newly defined cutoff values of CEA level.

Therefore, this study aimed to define the optimal cutoff value of CEA level in the perioperative period and to investigate the clinical impact of trajectory changes in CEA level measured during the immediate postoperative period in patients with CRC.

## Methods

### Patients

This was a retrospective, single-institution study that included patients who were diagnosed with stage I–III CRC who underwent curative surgery at Gangnam Severance Hospital, Yonsei University College of Medicine, between January 2004 and April 2014.

A total of 1751 patients who underwent surgery during the study period were initially selected. The inclusion criteria were as follows (1): histologically confirmed stage I–III adenocarcinoma of the colon and rectum and (2) available CEA-pre and CEA-post data. The exclusion criteria were as follows (1): stage 0, stage IV, or unknown stage (2); tumors located in the appendix or anus, or unknown tumor location (3); neuroendocrine tumor, gastrointestinal stromal tumor, or other types of carcinoma (4); preoperative chemoradiotherapy or chemotherapy (5); emergency surgery (6); hereditary CRC, ulcerative colitis, or Crohn’s disease-associated cancer; and (7) double primary cancers. As a result, 1019 patients with available CEA-pre and CEA-post data remained eligible and were included in this study.

All procedures performed in this study were in accordance with the ethical standards of the institutional and/or national research committees and with the 1964 Declaration of Helsinki and its later amendments or comparable ethical standards. This study was approved by the institutional review board of Gangnam Severance Hospital, Yonsei University College of Medicine, which waived the requirement for informed consent from patients owing to the retrospective study design.

### Measured Outcomes and Definitions of CEA-Pre, CEA-Post, CEA-Delta and CEA-Trend

The analyzed variables were sex, age, body mass index (BMI), tumor location, complications, histologic grade, lymphovascular invasion (LVI), tumor stage, chemotherapy, and CEA level. CEA-pre was usually measured within 31 days before surgery. CEA-post was defined as CEA level measured 4–9 days postoperatively. CEA-delta was calculated as CEA-post minus CEA-pre. The optimal cutoff values for classifying whether the CEA levels were low or high for predicting OS were set using the X-tile program (20). The patients were divided into three groups according to CEA-trend, as follows: normal, low CEA-pre; normalized, high CEA-pre/low CEA-post; and elevated, high CEA-pre/high CEA-post.

### CEA Measurement Method

The CEA immunoassay analyzer used for CEA measurement in our hospital was once changed during the study period. Roche MODULAR ANALYTICS E170 (Roche Diagnostics GmbH, Mannheim, Germany), a electrochemiluminescence immunoassay (ECLIA) equipment, was used to measure CEA level until replaced by Beckman-Coulter UniCel DxI 800 (Beckman Coulter inc., Brea,CA, USA), a chemiluminescence immunoassay (CLIA) equipment, at 2008.12.12.

### Evaluation of Prognostic Role of CEA-Trend Depending on the Date of CEA Analyzer Change

In order to check whether there is a difference in the usefulness of the prognostic model according to the change of the CEA measurement equipment, we divided the study period into two periods (period 1 and periods 2) based on the date when the analyzer was changed, and according to each sub period, the Kaplan-Meier survival curve and iAUC comparisons between CEA-trend with conventional classification by CEA level 5ng/mL were independently performed according to the two periods.

### Follow-Up of Patients

The patients in our cohort had a median follow-up period of 94 months (interquartile range [IQR] 72–126 months). They were followed up at 3–6-month intervals for 2–3 years and at 6-month intervals up to 5 years. The CEA level was checked at each follow-up point, mostly constant at 3 months. If the CEA level increased from the previous result, further imaging studies were performed to check for recurrence according to the physician’s decision.

### Statistical Analyses

OS was defined as the time from the date of surgery to the date of death of any cause or the last follow-up. The Kaplan–Meier method and log-rank test were used to analyze the dependence of OS on CEA-pre, CEA-post, CEA-delta, and CEA-trend. Univariate and multivariate analyses of OS were conducted using Cox proportional hazard regression models to determine the HRs and 95% CIs according to different variables. Various clinical predictors expected to independently influence OS were first tested in the univariate analysis, and variables that showed statistical significance (*p*<.05) were entered into the multivariate analysis.

The prognostic predictive abilities of CEA-pre, CEA-post, CEA-delta, and CEA-trend were estimated and compared using the integrated area under the curve (iAUC). Differences in predictive ability between each CEA category were calculated using the bootstrapping method.

Statistical significance was set at *p*<.05. All statistical analyses were performed using R (version 3.6.3; R-project, Institute for Statistics and Mathematics, Vienna, Austria).

## Results

A total of 1019 patients were included in this study. The median (IQR) values of CEA-pre and CEA-post were 2.9 (1.7–5.7) and 1.7 (1.0–2.6) ng/mL, respectively. The median interval between surgery and the test for CEA-post was 7 days. During the study period, 250 patients (24.5%) died.

### Optimal Cutoff Values of CEA-Related Parameters

The optimal cutoff value of both CEA-pre and CEA-post in association with OS was determined to be 2.3 ng/mL. The patients were subsequently categorized into two groups according to the cutoff value. Of the patients, 362 (35.5%) and 685 (67.2%) were allocated to the low CEA-pre and low CEA-post groups, respectively. The optimal cutoff value of CEA-delta was determined to be -0.93 ng/mL (**Supplementary Figure 1**).

### Comparison of Patient Characteristics Between the Low and High CEA Groups

Significant differences were found in age (*p*<.001), tumor size (*p*=.004), complications (*p*<.007), LVI (*p*=.002), stage (*p*<.001), and chemotherapy (*p*=.009) between the low CEA-pre and high CEA-pre groups, whereas sex (*p*=.103), BMI (*p*=.397), smoking (*p*=.241), tumor location (*p*=.327), histologic grade (*p*=.652), and number of retrieved lymph nodes (*p*=.332) were not significantly different between the two groups. In contrast, between the low and high CEA-post groups, age (*p*=.009), smoking (*p*=.018), tumor size (*p*<.001), LVI (*p*=.003), stage (*p*<.001), and chemotherapy (*p*=.020) were significantly different. The two groups showed no significant differences in sex (*p*=.521), BMI (*p*=.990), tumor location (*p*=.151), complications (*p*=.056), histologic grade (*p*=.275), and number of retrieved lymph nodes (*p*=.730) (**Table 1**).

**Table 1:** Patient characteristics according to CEA.

		Preoperative CEA			Postoperative CEA		
		Low (n = 362)	High (n = 657)	*p*	Low (n = 685)	High (n = 334)	*p*
Sex	Female	167 (46.1)	267 (40.6)		297 (43.4)	137 (41)	
	Male	195 (53.9)	390 (59.4)	0.103	388 (56.6)	197 (59)	0.521
Age (years)	< 65	232 (64.1)	299 (45.5)		377 (55)	154 (46.1)	
	≥ 65	130 (35.9)	358 (54.5)	<0.001	308 (45)	180 (53.9)	0.009
BMI (kg/m^2^)	Mean (SD)	23.7 (3)	23.5 (3.2)	0.397	23.6 (3)	23.6 (3.2)	0.990
Smoking	No	262 (72.4)	451 (68.6)		496 (72.4)	217 (65)	
	Yes	100 (27.6)	206 (31.4)	0.241	189 (27.6)	117 (35)	0.018
Tumor location	Rt. Colon	92 (25.4)	167 (25.4)		166 (24.2)	93 (27.8)	
	Lt. Colon	165 (45.6)	272 (41.4)		308 (45)	129 (38.6)	
	Rectum	105 (29)	218 (33.2)	0.327	211 (30.8)	112 (33.5)	0.151
Tumor size (cm)	< 5	240 (66.3)	374 (56.9)		444 (64.8)	170 (50.9)	
	≥ 5	122 (33.7)	283 (43.1)	0.004	241 (35.2)	164 (49.1)	<0.001
Complications	No	294 (81.2)	483 (73.5)		535 (78.1)	242 (72.5)	
	Yes	68 (18.8)	174 (26.5)	0.007	150 (21.9)	92 (27.5)	0.056
Histologic grade	G1 & G2	338 (93.4)	607 (92.4)		640 (93.4)	305 (91.3)	
	G3 & MC & SRC	24 (6.6)	50 (7.6)	0.652	45 (6.6)	29 (8.7)	0.275
LVI	Absent	257 (71)	398 (60.6)		464 (67.7)	191 (57.2)	
	Present	57 (15.7)	156 (23.7)		133 (19.4)	80 (24)	
	unknown	48 (13.3)	103 (15.7)	0.002	88 (12.8)	63 (18.9)	0.003
Retrieved LNs	< 12	53 (14.6)	113 (17.2)		114 (16.6)	52 (15.6)	
	≥ 12	309 (85.4)	544 (82.8)	0.332	571 (83.4)	282 (84.4)	0.730
Stage	I	126 (34.8)	119 (18.1)		198 (28.9)	47 (14.1)	
	II	117 (32.3)	233 (35.5)		233 (34)	117 (35)	
	III	119 (32.9)	305 (46.4)	<0.001	254 (37.1)	170 (50.9)	<0.001
Chemotherapy	No	151 (41.7)	219 (33.3)		260 (38.8)	104 (31.1)	
	Yes	211 (58.3)	438 (66.7)	0.009	419 (61.2)	230 (68.9)	0.020

BMI, Body mass index; CEA, Carcinoembryonic antigen; MC, Mucinous adenocarcinoma; SRC, Signet-ring cell; LVI, Lymphovascular invasion; LN, Lymph node.

SD, Standard Deviation.

### Kaplan–Meier Curve Analysis According to CEA-Pre, CEA-Post, CEA-Delta, and CEA-Trend

**Figure 1:**
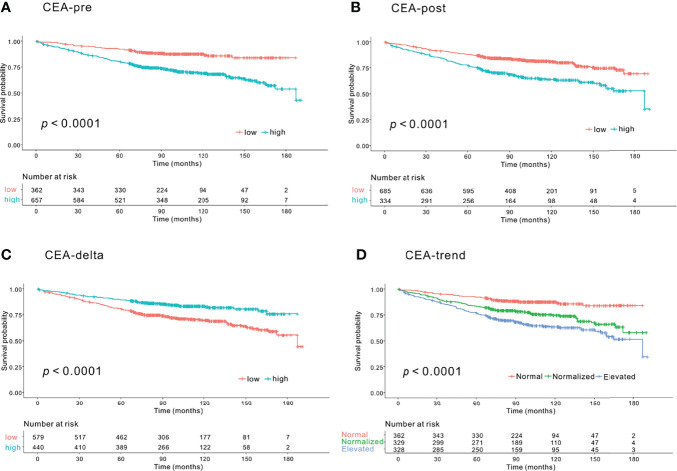
Kaplan–Meier survival curve analysis of groups according to carcinoembryonic antigen (CEA) level for overall survival (OS). **(A)** Preoperative CEA (CEA-pre). The low CEA-pre group (CEA-pre <2.3 ng/mL) showed better OS than the high CEA-pre group (CEA-pre <2.3 ng/mL) (5-year OS: 92.2% vs. 80.4%, *p*<.0001). **(B)** Early postoperative CEA (CEA-post). The low CEA-post group (CEA-post <2.3 ng/mL) showed better OS than the high CEA-post group (CEA-post <2.3 ng/mL) (5-year OS: 87.9% vs. 77.9%, *p*<.0001). **(C)** CEA level change (CEA-delta). The low CEA-delta group (CEA-delta <-0.93 ng/mL) had worse OS than the high CEA-delta group (CEA-delta <-0.93 ng/mL) (5-year OS: 80.8% vs. 89.5%, *p*<.0001). **(D)** CEA-trend. The normal CEA group (low CEA-pre group) had the best OS followed by the normalized (high CEA-pre and low CEA-post group) and elevated CEA groups (high CEA-pre and high CEA-post groups) (5-year OS: 92.2% vs. 83.5% vs. 77.2%, *p*<.0001).

Significant differences were observed in 5-year OS between the low and high groups based on CEA-pre (92.2% vs. 80.4%, *p*<.0001) (**Figure 1** **A**), CEA-post (87.9% vs. 77.7%, *p*<.0001) (**Figure 1** **B**), and CEA-delta (80.8% vs. 89.5%, *p*<.0001) (**Figure 1C**). In addition, the three groups classified according to CEA-trend showed significant differences in 5-year OS (92.2% in the normal group vs. 83.5% in the normalized group vs. 77.2% in the elevated group, *p*<.0001) (**Figure 1** **D**).

### Univariate and Multivariate Analyses of OS

In univariate Cox regression analysis, sex (HR, 1.296; 95% CI, 1.001–1.676; *p*=.048), age (HR, 2.432; 95% CI, 1.876–3.167; *p*<.001), complications (HR, 1.673; 95% CI, 1.286–2.177; *p*<.001), LVI (HR, 1.681; 95% CI, 1.252–2.257; *p*<.001), stage (stage II vs. I [HR, 1.855; 95% CI, 1.218–2.827; *p*=.004], stage III vs. I [HR, 3.152; 95% CI, 2.125–4.677; *p*<.001]), chemotherapy (HR, 0.771; 95% CI, 0.596–0.996; *p*=.047), CEA-pre (HR, 2.687; 95% CI, 1.946–3.712; *p*<.001), CEA-post (HR, 2.045; 95% CI, 1.595–2.622; *p*<.001), CEA-delta (HR, 0.509; 95% CI, 0.387–0.669; *p*<.001), and CEA-trend (normalized vs. normal [HR, 2.175; 95% CI, 1.516–3.119; *p*<.001], elevated vs. normal [HR, 3.241; 95% CI, 2.299–4.570, *p*<.001]) showed significant associations with OS (**Table 2**).

**Table 2:** Univariate and multivariate analysis of factors associated with overall survival.

Variables	Categorization	Univariate analysis		Multivariate analysis	
		HR (95% CI)	*p*	HR (95% CI)	*p*
Sex	Female	1		1	
	Male	1.296 (1.001–1.676)	0.048	1.213 (0.935–1.573)	0.144
Age (years)	< 65	1		1	
	≥ 65	2.432 (1.867–3.167)	<0.001	1.814 (1.373–2.396)	<0.001
BMI (kg/m^2^)	< 25	1			
	≥ 25	0.784 (0.592–1.038)	0.088		
Smoking	No	1			
	Yes	1.225 (0.941–1.594)	0.131		
Tumor location	Rt. Colon	1			
	Lt. Colon	0.853 (0.626–1.161)	0.312		
	Rectum	0.922 (0.668–1.273)	0.623		
Complications	No	1		1	
	Yes	1.673 (1.286–2.177)	<0.001	1.539 (1.180–2.007)	0.001
Histologic grade	G1 & G2	1			
	G3 & MC & SRC	1.419 (0.923–2.180)	0.110		
LVI	Absent	1			
	Present	1.681 (1.252–2.257)	<0.001		
	No data	1.261 (0.897–1.773)	0.180		
Stage	I	1		1	
	II	1.855 (1.218–2.827)	0.004	2.417 (1.550–3.768)	<0.001
	III	3.152 (2.125–4.677)	<0.001	4.946 (3.152–7.762)	<0.001
Chemotherapy	No	1		1	
	Yes	0.771 (0.596–0.996)	0.047	0.424 (0.313–0.574)	<0.001
CEA-pre (ng/mL)	Low	1			
	High	2.687 (1.946–3.712)	<0.001		
CEA-post (ng/mL)	Low	1			
	High	2.045 (1.595–2.622)	<0.001		
CEA-delta (ng/mL)	Low	1			
	High	0.509 (0.387–0.669)	<0.001		
CEA-trend	Normal	1		1	
	Normalized	2.175 (1.516–3.119)	<0.001	1.838 (1.277–2.645)	0.001
	Elevated	3.241 (2.299–4.570)	<0.001	2.412 (1.701–3.421)	<0.001

HR, Hazard Ratio; CI, Confidence Interval.

BMI, Body mass index; CEA, Carcinoembryonic antigen; MC, Mucinous adenocarcinoma; SRC, Signet-ring cell; LVI, Lymphovascular invasion.

In multivariate Cox regression analysis, age (HR, 1.814; 95% CI, 1.373–2.396; *p*<.001), complications (HR, 1.539; 95% CI, 1.180–2.006; *p*=.001), stage (stage II vs. I [HR, 2.417; 95% CI, 1.550–3.768; *p*<.001], stage III vs. I [HR, 4.947; 95% CI, 3.152–7.763; *p*<.001]), chemotherapy (HR, 0.424; 95% CI, 0.313–0.574; *p*<.001), and CEA-trend (normalized vs. normal [HR, 1.838; 95% CI, 1.277–2.645; *p*=.001], elevated vs. normal [HR, 2.412; 95% CI, 1.701–3.421; *p*<.001]) were independent risk factors for OS (**Table 2**).

### iAUC Comparison

**Figure 2:**
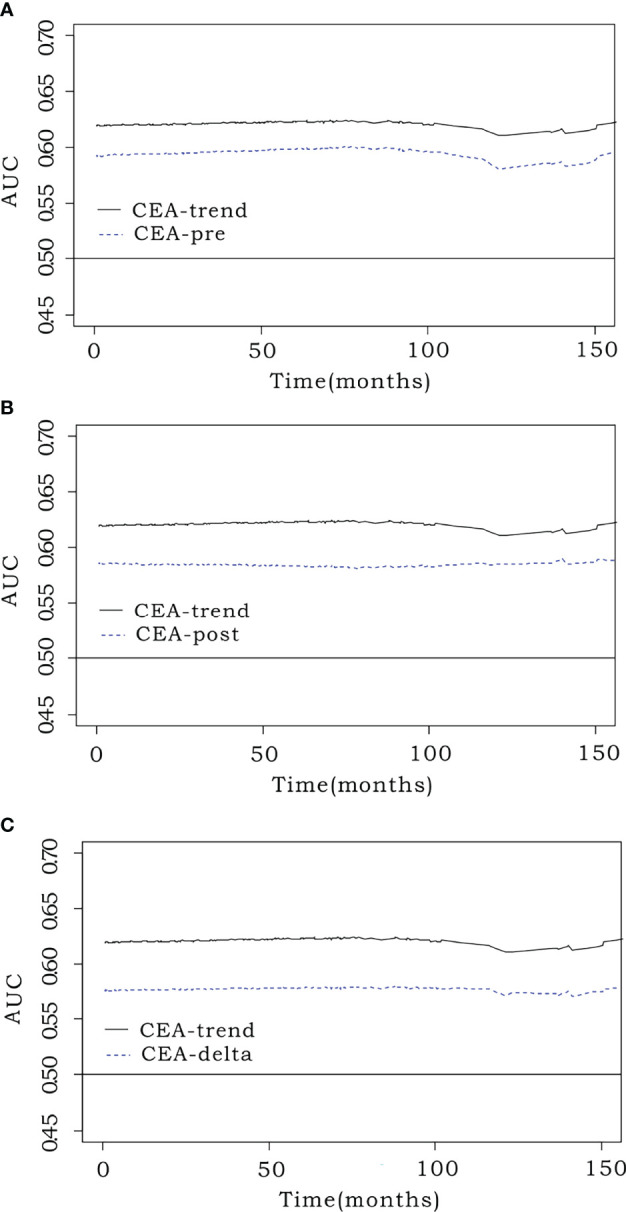
Comparison of predictive performance using the integrated area under the curve (iAUC). Prognostic efficacy was measured using the iAUC (weighted mean of AUCs over the follow-up period) for each model. The time-dependent receiver operating characteristic curve of CEA-trend (0.62; 95% confidence interval [CI], 0.571–0.675) had an elevated position over that of **(A)** preoperative CEA (CEA-pre) (0.583; 95% CI, 0.513–0.636) (bootstrap iAUC mean difference=0.037; 95% CI, 0.009–0.116), **(B)** early postoperative CEA (CEA-post) (0.591; 95% CI, 0.541–0.655) (bootstrap iAUC mean difference=0.029; 95% CI, 0.012–0.052), and **(C)** CEA level change (CEA-delta) (0.568; 95% CI, 0.504–0.616) (bootstrap iAUC mean difference=0.052; 95% CI, 0.017–0.116).

The iAUC value of CEA-trend (0.620; 95% CI, 0.571–0.675) was higher than that of CEA-pre (0.583; 95% CI, 0.513–0.636) (bootstrap iAUC mean difference=0.037; 95% CI, 0.009–0.116), CEA-post (0.591; 95% CI, 0.541–0.655) (bootstrap iAUC mean difference=0.029; 95% CI, 0.012–0.052), and CEA-delta (0.568; 95% CI, 0.504–0.616) (bootstrap iAUC mean difference=0.052; 95% CI, 0.017–0.116) (**Figure 2**).

### Kaplan–Meier Survival Curve Analysis of CEA-Trend According to CRC Stage

**Figure 3:**
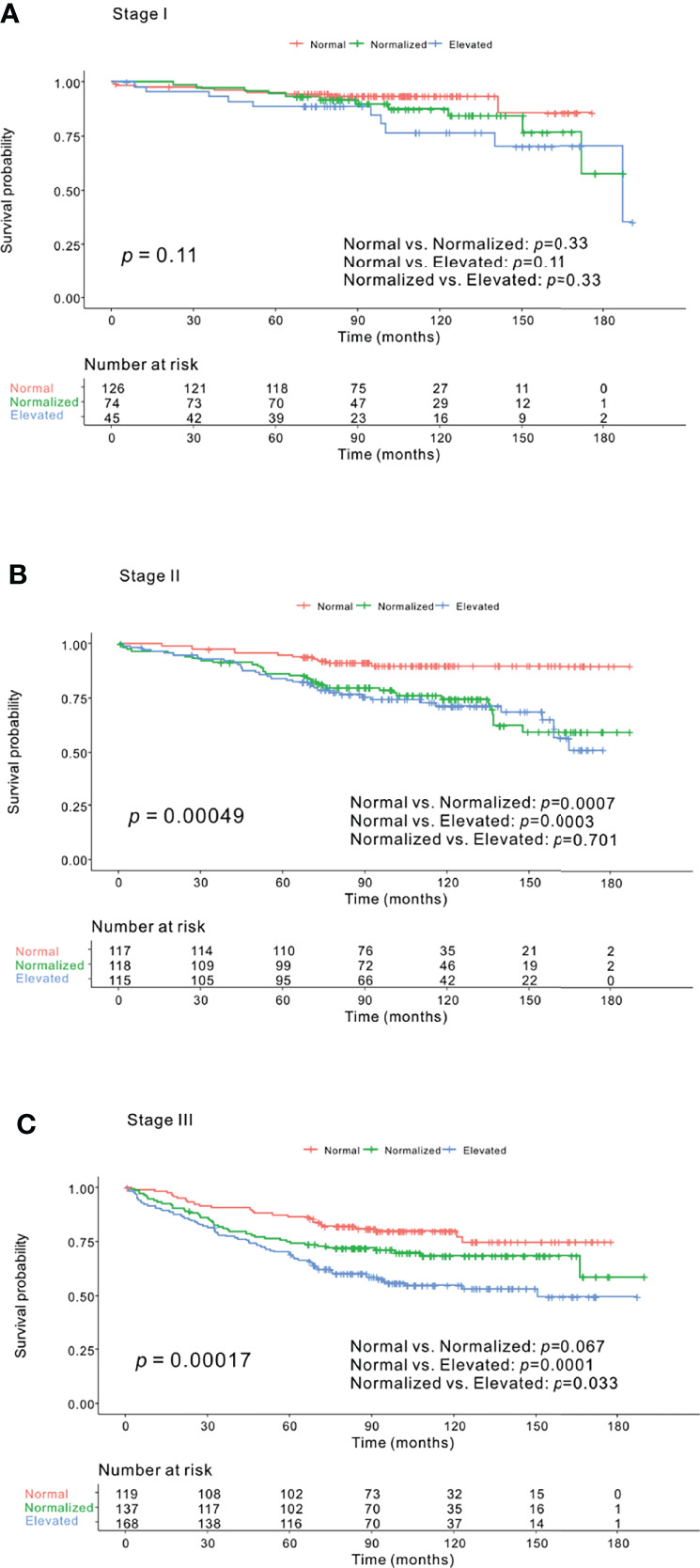
Kaplan–Meier survival curve analysis of carcinoembryonic antigen (CEA)-trend according to colorectal cancer (CRC) stage. **(A)** Stage I CRC. No significant distinction in overall survival (OS) was found among the normal, normalized, and elevated groups (*p*=.11). **(B)** Stage II CRC. The normal group had better OS than both the normalized (*p*=.0007) and elevated (*p*=.0003) groups, although no significant difference was found in OS between the normalized and elevated groups (*p*=.701) (*p*=.00049). **(C)** Stage III CRC. The normal and normalized groups did not show a difference in OS (*p*=.067), whereas the normal (*p*=.0001) and normalized groups (*p*=.033) were associated with better OS than the elevated group (*p*=.00017).

No significant difference in OS was found between the normal, normalized, and elevated CEA groups among patients with stage I CRC (*p*=.11). In patients with stage II CRC, there was a significant difference in OS. The elevated (*p*=.0003) and normalized groups (*p*=.0007) had worse survival probability than the normal group. The normalized group did not show a significant difference (*p*=.701) from the elevated group. In patients with stage III CRC, the elevated group had worse survival than the normal (*p*=.0001) and normalized (*p*=.033) groups. No significant difference in OS was detected between the normal and normalized groups (*p*=.067) (**Figure 3**).

### Kaplan–Meier Survival Curve Analysis of CEA-Trend in Patients With CEA-Pre <5 ng/mL (n=732)

A total of 732 patients showed a CEA-pre of <5 ng/mL, and these patients would be classified into the “normal group” according to the criteria used in previous studies (5, 7, 8, 10–12). Among them, 362, 280, and 90 patients were classified into the normal, normalized, and elevated groups based on the CEA-trend classification in the present study. A significant difference in 5-year OS was observed between the normal and normalized groups (92.2% vs. 83.4%, *p*<.001). The elevated group showed worse 5-year OS than the normal group (76.7% vs. 92.2%, *p*<.001), but showed a similar OS to the normalized group (76.7% vs. 83.4%, *p*=.067) (**Supplementary Figure 4**).

### iAUC Comparison Between CEA-Trend and Conventional Classification Using the Cutoff Value of 5 ng/mL in the Whole Cohort (n=1019)

**Figure 4:**
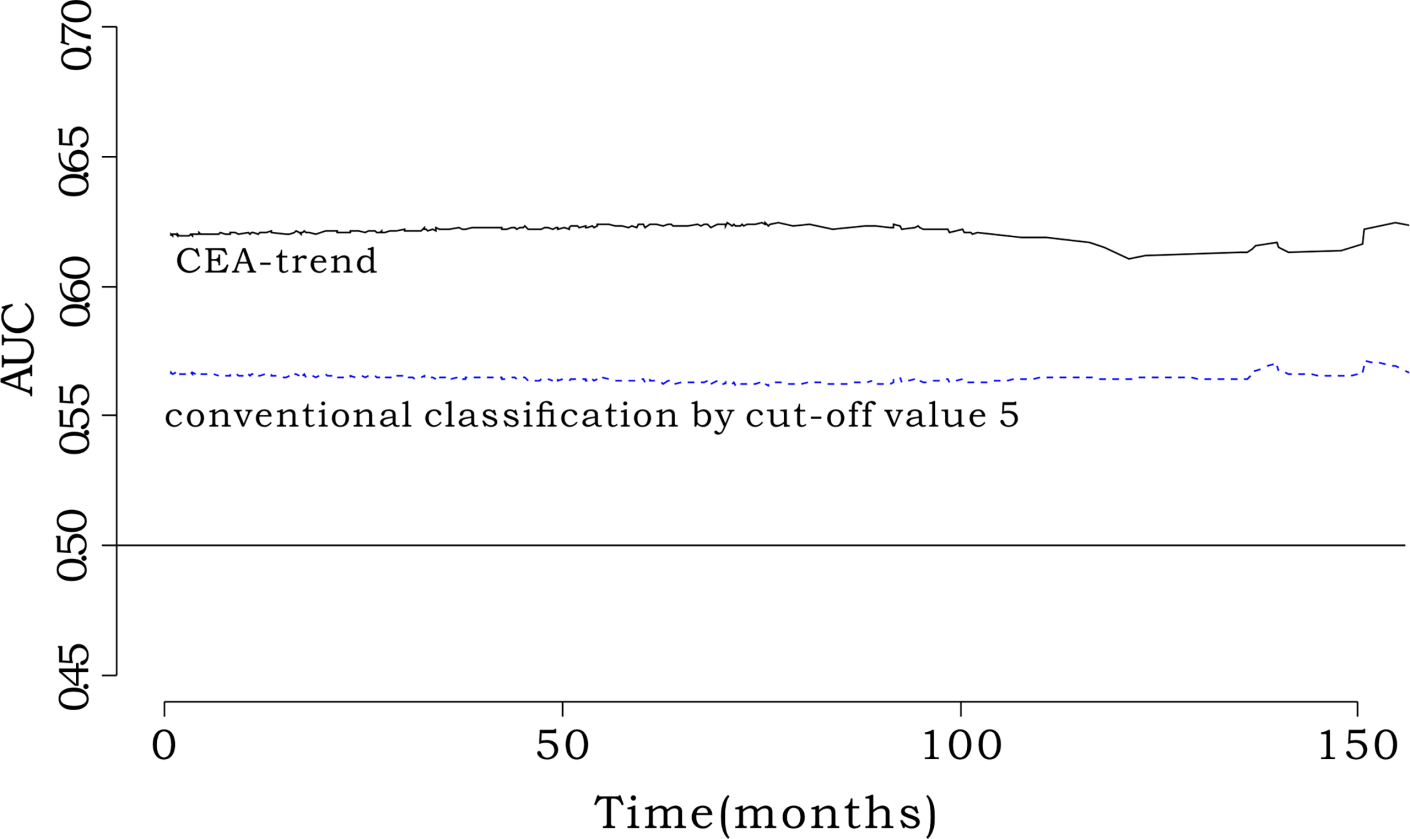
Comparison of iAUC between CEA-trend and conventional classification by cut-off value 5. The integrated AUC value of CEA-trend (0.619; 95% confidence interval [CI], 0.553-0.668) was higher than that of the conventional classification by cut-off value 5 (0.570, 95% CI: 0.514-0.634) (bootstrap iAUC mean difference=0.048, 95% CI=0.002-0.085). “conventional classification by cut-off value 5” composed of stratification by the cut-off value 5 such as “Group 1: preoperative CEA<5, Group 2: preoperative CEA≥5 & postoperative CEA<5, and Group 3: preoperative CEA≥5 & postoperative CEA≥5”.

We defined groups according to the conventional classification using the cutoff value of 5 ng/mL, as follows: group 1, CEA-pre ≤5 ng/mL; group 2, CEA-pre >5 ng/mL and CEA-post ≤5 ng/mL; and group 3, CEA-pre >5 ng/mL and CEA-post >5 ng/mL. The iAUC value of CEA-trend was higher than that of the conventional classification using a cutoff value of 5 ng/mL (0.571; 95% CI, 0.517–0.628) (bootstrap iAUC mean difference=0.047; 95% CI, 0.008–0.082) (**Figure 4**).

### Prognostic Impact of CEA-Trend According to the Different Time Periods Depending on the CEA Analyzer Change

In Kaplan-Meier survival curve, the CEA-trend could stratify patients’ survival irrespective of time periods dichotomized by the analyzer change date (**Supplementary Figure 5**). Also, iAUC of CEA-trend was higher than that of conventional classification by cut-off value 5 in both period 1 and period 2 (**Supplementary Figure 6**).

## Discussion

This study demonstrated that a cutoff value of 2.3 ng/mL for both CEA-pre and CEA-post could discriminate the OS of patients. Furthermore, the trajectory change according to the combined stratification using CEA-pre and CEA-post could provide better prognostic performance in comparison with either CEA-pre alone or CEA-post alone in patients with nonmetastatic CRC. In detail, the normalized group showed worse OS than the normal group in patients with stage II CRC, whereas there was no difference in OS between the normalized and normal groups in patients with stage III CRC. Thus, the clinical effect of CEA changes may differ according to the CRC stage.

Although most previous studies assessed CEA-pre or CEA-post individually rather than in combination, several studies have investigated the clinical impact of the trajectory change of CEA level in patients with CRC. However, the results showed some discrepancies across studies, especially between the normal and normalized groups (15, 16). In addition, Kim et al. reported that colon cancer patients with elevated CEA levels showed worse disease-free survival (DFS) or OS than the normal or normalized groups (21). In their study, the survival difference between the normal and normalized groups was evident in OS alone, but not in DFS. Therefore, it appears that there are some difficulties in using the trajectory change of CEA level during the perioperative period in making important clinical decisions in patients with CRC.

Compared with previous studies considering the trajectory change of CEA level, our analysis had a unique design and novel results. First, most previous studies used CEA levels measured within 12 weeks after surgery (15, 16, 21, 22). Although the 12-week interval is considered one of the acceptable time periods between surgery and chemotherapy initiation, some guidelines and studies recommend an interval of 8 weeks before initiating adjuvant chemotherapy in patients with CRC (23–26). Furthermore, several studies reported initiating adjuvant chemotherapy approximately 4–5 weeks or even <3 weeks after surgery (27–29). In this regard, when adopting CEA change in determining whether to start chemotherapy within 8 weeks after surgery, data on CEA levels measured within 12 weeks after surgery might inevitably have some limitations. In contrast, our results can be universally applied in clinical practice because the measurement time point was relatively constant. Second, previous studies applied the conventionally recommended value of 5 ng/mL as the cutoff value and did not identify any optimal cutoff values. Considering the newly proven lower optimal cutoff values of CEA in several studies, (17–19) we attempted to determine optimal cutoff values with respect to CEA-pre and CEA-post. Interestingly, the meaningful value for discrimination was the same for CEA-pre and CEA-post. Third, the normalized group in our analysis showed worse prognosis than the normal group in the whole patient population (*p*<.001). However, when we performed subgroup analysis according to CRC stage, different trends were observed. In patients with stage II CRC, the normalized group showed worse OS than the normal group (*p*=.0007), whereas the OS showed no difference between the normalized and normal groups in patients with stage III CRC (*p*=.067). The exact reason for these different prognoses depending on the CRC stage could not be explained in this study. However, in the previous two studies, the survival of the normalized group in the study including colon cancer patients was similar to the normal group (16), whereas another study including rectal cancer patients only, the survival of the normalized group was lower than the normal group (15), suggesting possible relation with our results. Fourth, the group with consistently high CEA levels in both the preoperative and early postoperative periods, despite the lower cutoff point, had a much worse survival than the others. Notably, among patients with a CEA-pre of <5 ng/mL, who would be classified into the normal group according to the criteria of previous studies, many were classified into the normalized or elevated group based on the criteria used in the present study. These normalized and elevated groups, despite having a CEA-pre of <5 ng/mL, showed worse OS than the normal group (**Supplementary Figure 4**). We further analyzed the discriminatory power of the criteria and observed that the iAUC value was significantly higher in the newly defined CEA-trend than in the conventional stratification using 5 ng/mL as the cutoff value. Because the elevated group might be candidates for more intensive postoperative follow-up or other kinds of chemotherapy regimens after surgery, further research is needed to determine whether our new classification for CEA can be a useful selection criterion for this purpose.

In the past, without modern imaging technologies such as computed tomography (CT) or magnetic resonance imaging (MRI), and positron emission tomography/CT, CEA-pre measurement used to have a significant role as a predictive factor suspecting distant metastasis in CRC patients. However, as time passed by, the role of CEA started to be replaced by advanced imaging modalities, with raising questions about the effectiveness of CEA-pre measurement. However, our study may provide new evidence on the potential clinical efficacy of CEA-pre and CEA-post measurements as important prognostic factors. Efforts to initiate postoperative adjuvant chemotherapy as early as possible are increasingly being adopted (30). Considering the requirement for robust biomarkers for adjuvant chemotherapy for high-risk patients with stage II or stage III CRC, our findings could offer a viable option for determining the timing of postoperative chemotherapy even in patients with early chemotherapy initiation. However, the real efficacy of these trajectory changes in determining postoperative chemotherapy and its clinical impact should be evaluated in a large-scale prospective study. Moreover, further investigations are needed to prove the hypothesis of this study.

Our study had some limitations. This study had a retrospective single-center design and thus might have inevitable selection bias. Although our study suggested 2.3 ng/mL as a new prognostic cutoff value for OS, there is no consensus on the cutoff values of CEA-related parameters. To enhance the general applicability of our findings, the clinical significance of trajectory CEA changes should be evaluated in different cohorts. In our study, as mentioned earlier, the time point of early postoperative laboratory examination was highly specified to minimize the effect of different time points of CEA measurement after surgery. However, the application of laparoscopic surgery, which is associated with enhanced postoperative recovery, could reduce the length of hospital stay. Thus, performing a CEA test in the early postoperative period, especially at <7 days, might not be a practical option in some centers. Considering the CEA half-life of 3–5 days, the adequate and clinically applicable date of constant CEA sampling needs to be investigated. Lastly, the analyzer used for CEA measurement have once been changed from Elecsys E170 to Unicel Dxi800 during the study period. The CEA value may vary slightly depending on the measuring instruments and this may adversely affect the determination of the adequate cut-off value. Although we demonstrated that CEA-trend could be used as a promising prognosticator compared with conventional classification using cut-off value 5ng/mL irrespective of time periods, these results cannot confidentially rule out the effect of different CEA analyzer. Therefore, additional research to find the CEA value that can overcome this discordance by measuring technique is essential.

In conclusion, the combination of CEA-pre and CEA-post synergistically improves the prognostic accuracy compared with CEA-pre alone or CEA-post alone. Patients with persistently high CEA levels during the perioperative period showed the worst prognosis in this study. In addition, the normalized group showed worse survival outcomes than the normal group; however, the effect might be dependent on the CRC stage. The clinical implications of CEA-based stratification as a suitable indication for adjuvant chemotherapy and as a guide to developing patient-specific follow-up programs need to be further investigated.

## Data Availability Statement

The datasets presented in this article are not readily available because the datasets analyzed in this study can be used after institutional review board approval owing to ethical and privacy restrictions. Requests to access the datasets should be directed to JK, ravic@naver.com.

## Ethics Statement

The studies involving human participants were reviewed and approved by Institutional Review Board of Gangnam Severance Hospital, Yonsei University College of Medicine. Written informed consent for participation was not required for this study in accordance with the national legislation and the institutional requirements.

## Author Contributions

Study concepts, JK. Study design, JK. Data acquisition, JK, J-HL, E-SC, HL, S-JS, EP, SB, and KL. Quality control of data and algorithms, JK. Data analysis and interpretation, JK. Statistical analysis, JK. Manuscript preparation, JK and YJ. Manuscript editing, JK and YJ. Manuscript review, all authors. All authors contributed to the article and approved the submitted version.

## Conflict of Interest

The authors declare that the research was conducted in the absence of any commercial or financial relationships that could be construed as a potential conflict of interest.

## Publisher’s Note

All claims expressed in this article are solely those of the authors and do not necessarily represent those of their affiliated organizations, or those of the publisher, the editors and the reviewers. Any product that may be evaluated in this article, or claim that may be made by its manufacturer, is not guaranteed or endorsed by the publisher.
