# Supplementary material for: Clinical Significance of Early Carcinoembryonic Antigen Change in Patients With Nonmetastatic Colorectal Cancer
Source: Front Oncol. 2022 May 9;12:739614. doi: 10.3389/fonc.2022.739614 (PMC9124957; doi:10.3389/fonc.2022.739614)
Supplement: Supplementary file 1 [file DataSheet_1.docx]

**Supplementary Files**

Clinical significance of early carcinoembryonic antigen change in patients with nonmetastatic colorectal cancer

| (A) CEA-pre |  |
| --- | --- |
| 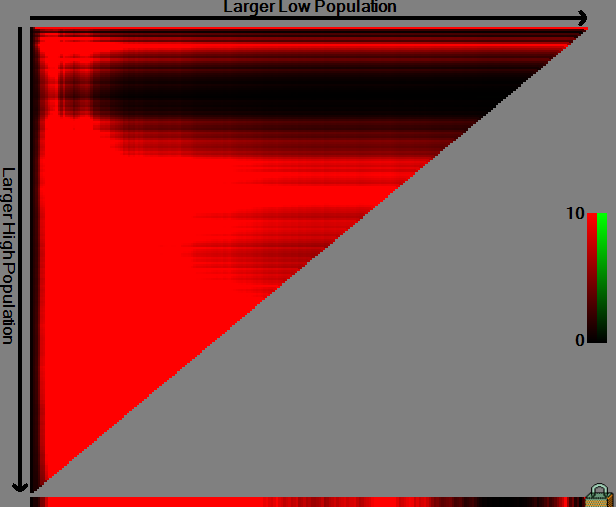 | 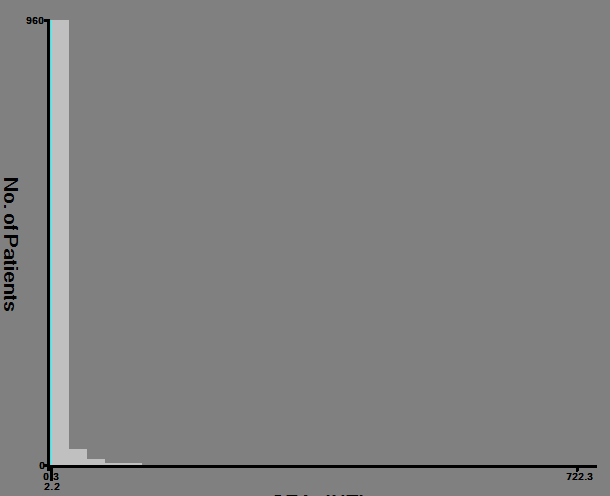 |
| (B) CEA-post |  |
| 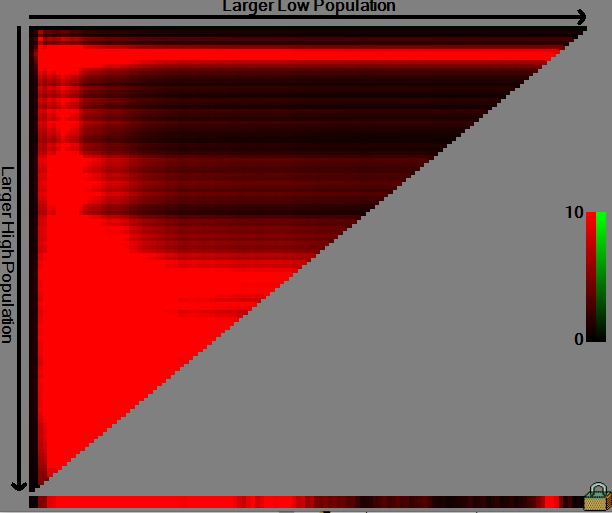 | 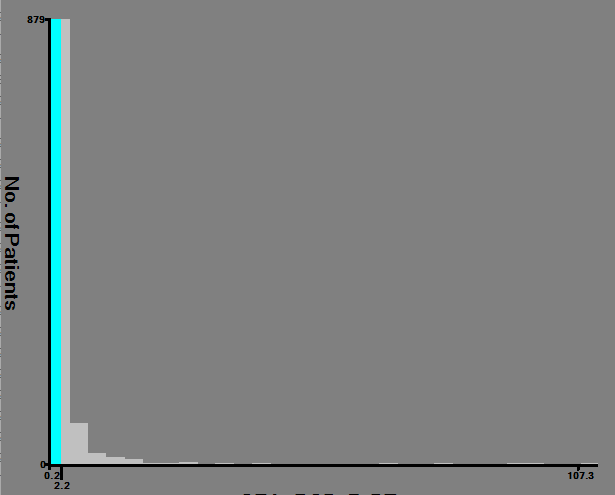 |
| (C) CEA-delta |  |
| 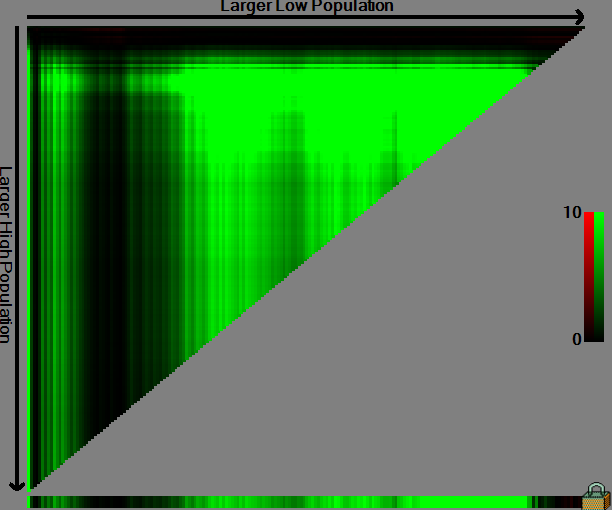 | 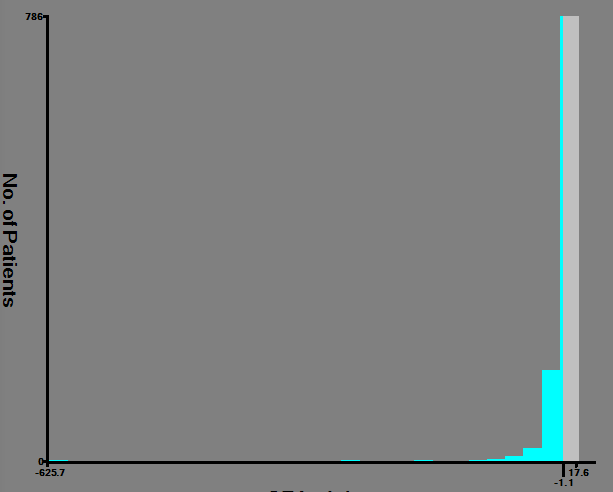 |

**Supplementary Figure 1. Defining optimal cut-off value of CEA-pre, CEA-post and CEA-delta using X-tile program.**

: According to the X-tile program, 2.3 ng/mL of CEA-pre (A), 2.3 ng/mL of CEA-post (B), and -0.93 ng/mL of CEA-delta (C) were defined as the optimal cut-off values respectively.


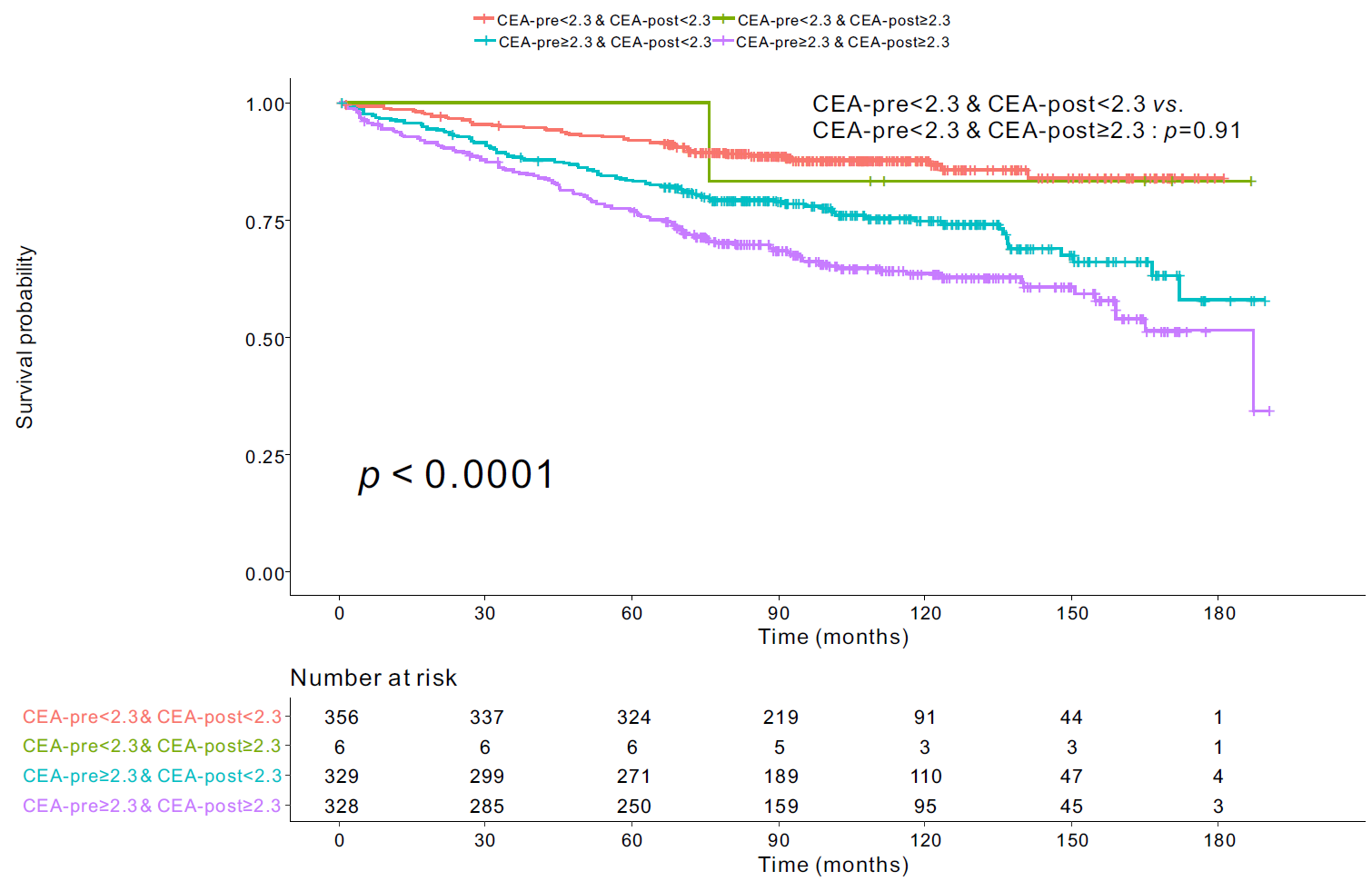


**Supplementary Figure 2. Kaplan-Meier survival curve of 4 groups composed of cut-off values of CEA-pre and CEA-post.**

: A total of 356, 6, 329 and 328 patients were allocated into CEA-pre<2.3 & CEA-post<2.3 group, CEA-pre<2.3 & CEA-post≥2.3 group, CEA-pre≥2.3 & CEA-post<2.3 group and CEA-pre≥2.3 & CEA-post≥2.3 group respectively. Among them, there was no survival difference between the CEA-pre<2.3 & CEA-post<2.3 group and the CEA-pre<2.3 & CEA-post≥2.3 groups (*p*=0.91).


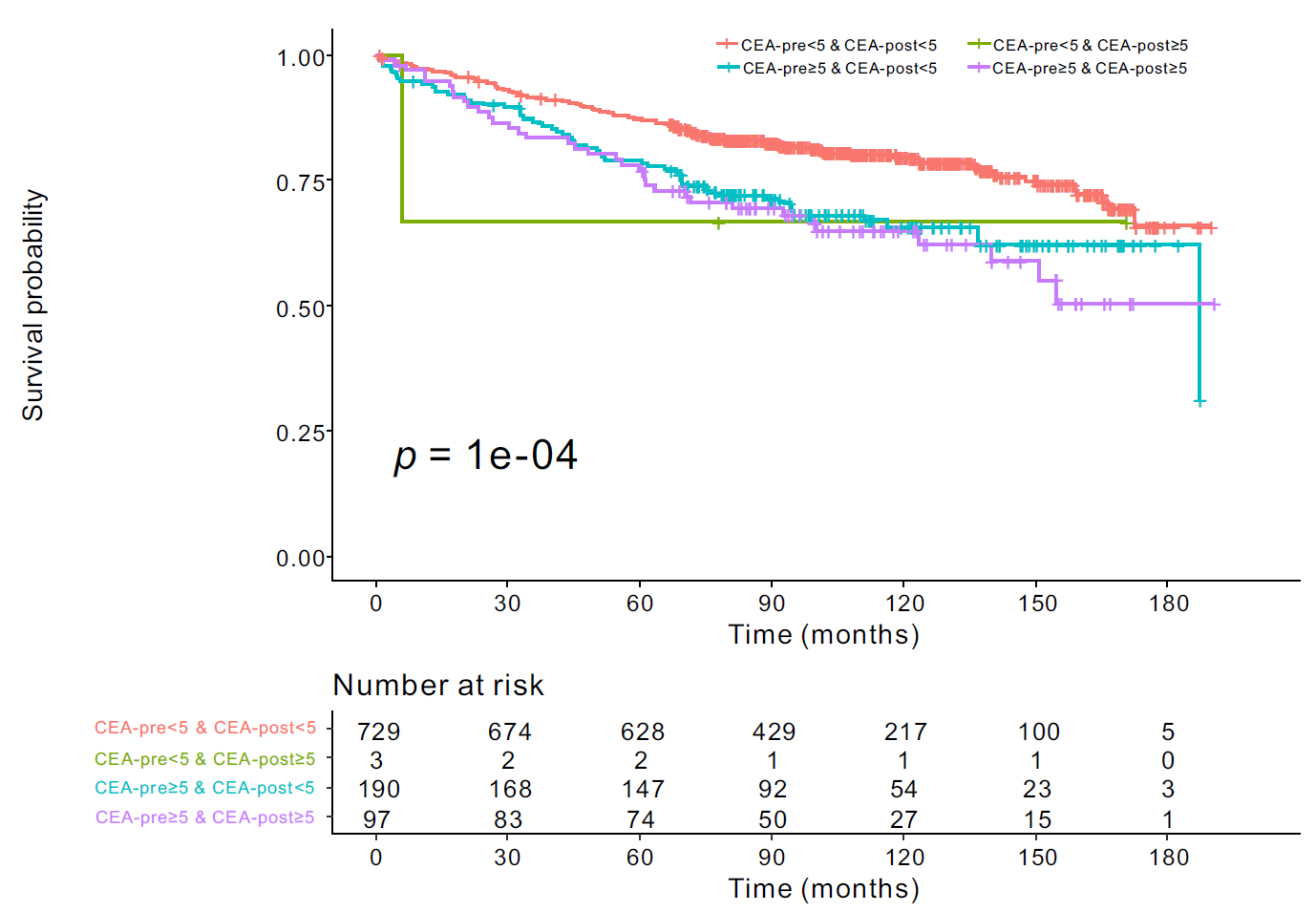


**Supplementary Figure 3. Kaplan-Meier survival curve of 4 groups according to the 5ng/dL stratification of CEA-pre and CEA-post.**

: A total of 729, 3, 190 and 97 patients were allocated into CEA-pre<5 & CEA-post<5 group, CEA-pre<5 & CEA-post≥5 group, CEA-pre≥5 & CEA-post<5 group and CEA-pre≥5 & CEA-post≥5 group respectively. Among them, there was no survival difference between the CEA-pre<5& CEA-post<5 group and the CEA-pre<5 & CEA-post≥5 groups (*p*=0.56).


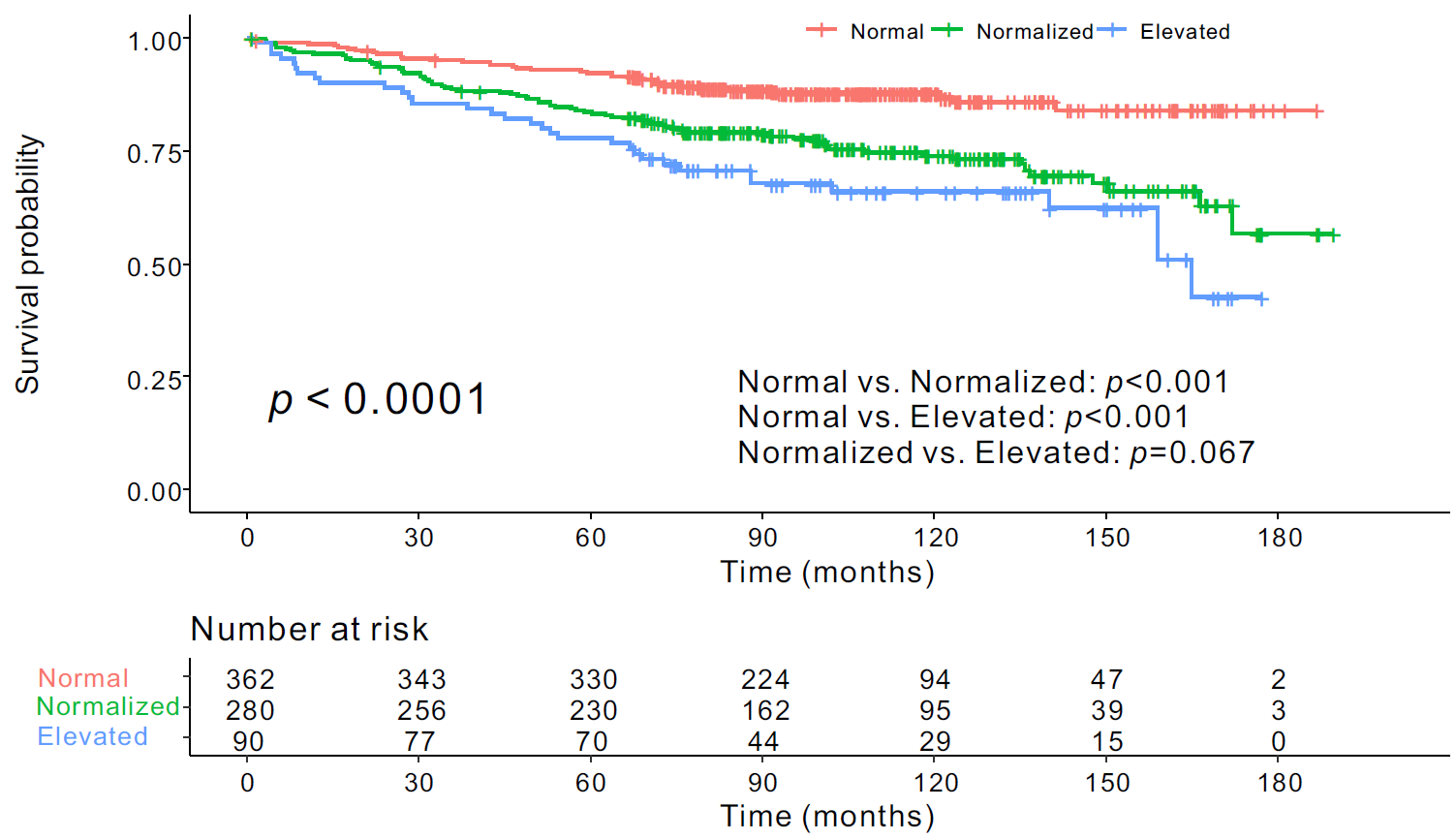


**Supplementary Figure 4. Kaplan–Meier survival curve analysis of carcinoembryonic antigen (CEA)-trend in patients with preoperative CEA level <5 ng/mL (n=732).**

: Among the 732 patients with a preoperative CEA level of <5 ng/mL, 362, 280, and 90 patients were classified into the normal, normalized, and elevated groups, respectively, according to the CEA-trend classification. A significant difference in 5-year overall survival (OS) was found between the normal and normalized groups (92.2% vs. 83.4%, *p*<.001). The elevated group showed worse 5-year OS than the normal group (76.7% vs. 92.2%, *p*<.001), but showed similar OS to the normalized group (76.7% vs. 83.4%, *p*=.067).

**Supplementary Table 1. Comparison of length of hospital stay among the surgery method according to open, laparoscopy and robot surgeries.**

|  | Open | Laparoscopy | Robot | *p* |
| --- | --- | --- | --- | --- |
| LOS [Mean, (SD)] (days) | 19.6 (15.9) | 8.3 (5.1) | 10.8 (7.6) | <0.001 |

LOS: Length of hospital stay

| (A) Period 1 |
| --- |
|  |
| (B) Period 2 |
|  |

**Supplementary Figure 5. Kaplan–Meier survival curve analysis of CEA-trend in patients according to the different period.**

: Kaplan-Meier survival curve showed significant different survival outcomes among CEA-trend in period 1 and 2 respectively.

Period 1: Jan 2004 - Dec 11 2008, period 2: Dec 12 2008 - April 2014

| (A) CEA-trend and conventional classification by cut-off value 5 in period 1 |
| --- |
| 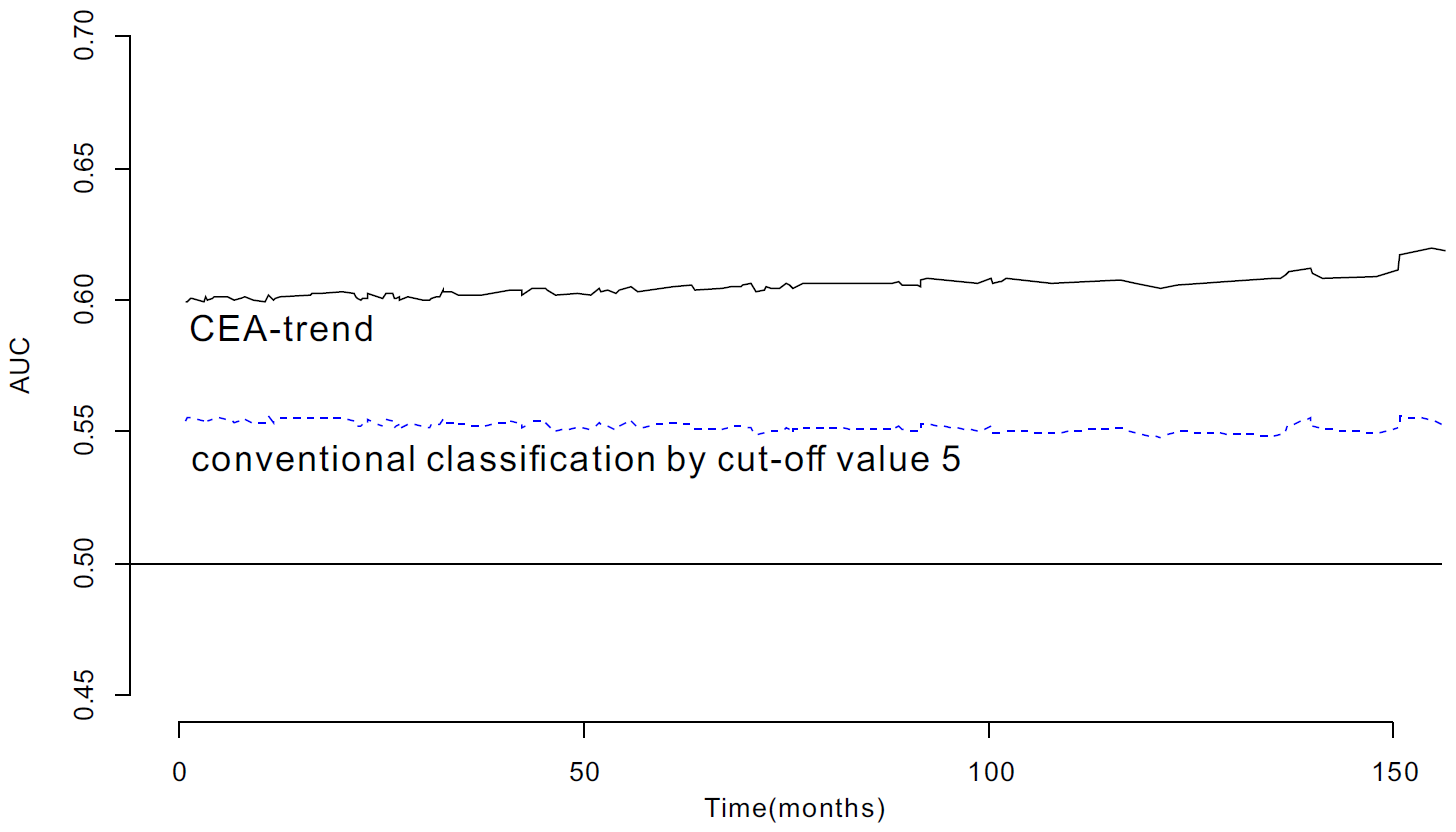 |
| (B) CEA-trend and conventional classification by cut-off value 5 in period 2 |
| 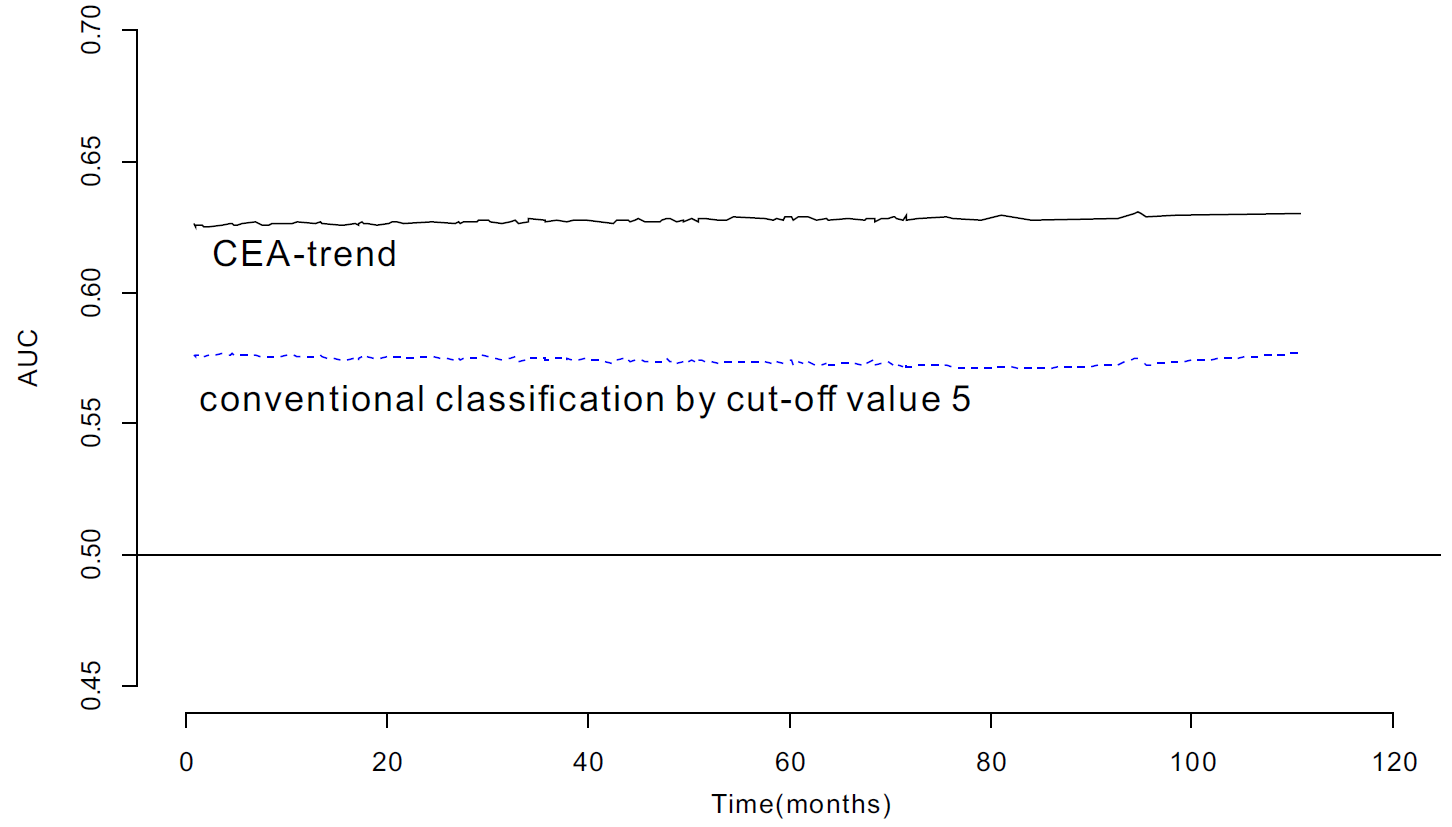 |

**Supplementary Figure 6. Comparison of integrated area under the curve (iAUC) between CEA-trend and conventional classification by cut-off value 5 according to the different period.**

: The integrated AUC value of CEA-trend (0.607; 95% confidence interval [CI], 0.546-0.666) was higher than that of the conventional classification by cut-off value 5 (0.558, 95% CI: 0.502-0.623) (bootstrap iAUC mean difference=0.048, 95% CI=0.002-0.099) in period 1.

The integrated AUC value of CEA-trend (0.627; 95% confidence interval [CI], 0.583-0.620) was higher than that of the conventional classification by cut-off value 5 (0.576, 95% CI: 0.535-0.620) (bootstrap iAUC mean difference=0.051, 95% CI=0.014-0.089) in period 2.

“conventional classification by cut-off value 5” composed of stratification by the cut-off value 5 such as “Group 1: preoperative CEA<5, Group 2: preoperative CEA≥5 & postoperative CEA<5, and Group 3: preoperative CEA≥5 & postoperative CEA≥5”.

Period 1: Jan 2004 - Dec 11 2008, period 2: Dec 12 2008 - April 2014
